# Efficacy of Minimally Invasive Trans-Sacral Canal Plasty between Patients with and without Failed Back Surgery Syndrome

**DOI:** 10.3390/medicina58020251

**Published:** 2022-02-07

**Authors:** Haruki Funao, Kimiaki Yokosuka, Junichi Ukai, Kazuo Nakanishi, Masaaki Paku, Takashi Tomita, Masahiro Hoshino, Takanori Saito, Ken Ishii, Koji Sato

**Affiliations:** 1 Department of Orthopaedic Surgery, School of Medicine, International University of Health and Welfare (IUHW), Narita 286-0048, Japan; 2 Department of Orthopaedic Surgery, International University of Health and Welfare (IUHW) Narita Hospital, Narita 286-8520, Japan; 3 Spine and Spinal Cord Center and Department of Orthopaedic Surgery, International University of Health and Welfare (IUHW), Mita Hospital, Tokyo 108-8329, Japan; 4 Department of Orthopedic Surgery, Kurume University School of Medicine, Kurume 830-0011, Japan; yokosuka_kimiaki@kurume-u.ac.jp; 5 Department of Orthopaedic Surgery, Japanese Red Cross Aichi Medical Center Nagoya Daini Hospital, Nagoya 466-8650, Japan; junukai9@nagoya2.jrc.or.jp; 6 Department of Orthopedics, Traumatology and Spine Surgery, Kawasaki Medical School, Kurashiki 701-0192, Japan; nakanishikazuo60@gmail.com; 7 Department of Orthopaedic Surgery, Kansai Medical University, Osaka 573-1191, Japan; kmu.orthopaedics.pak@gmail.com (M.P.); saitot@hirakata.kmu.ac.jp (T.S.); 8 Department of Orthopedic Surgery, Aomori Prefectural Central Hospital, Aomori 030-8553, Japan; tomming@arion.ocn.ne.jp; 9 Department of Orthopaedic Surgery, Sonoda Medical Institute Tokyo Spine Center, Tokyo 121-0807, Japan; mh-spine@mub.biglobe.ne.jp

**Keywords:** minimally invasive spinal treatment (MIST), minimally invasive spine surgery (MISS), intra-spinal canal treatment (ISCT), percutaneous epidural neuroplasty (PEN), trans-sacral canal plasty (TSCP)

## Abstract

*Background and Objectives:* Clinicians are required to manage a growing number of elderly patients with several medical comorbidities, and invasive surgical treatments are sometimes not advisable for these patients. The aim of this study was to evaluate the efficacy of minimally invasive intraspinal canal treatment, trans-sacral canal plasty (TSCP), for patients with and without failed back surgery syndrome (FBSS). *Materials and Methods:* A multicenter analysis was conducted. TSCP was performed in patients with chronic low back pain and leg pain due to lumbar spinal disorders. An adhesiolysis by TSCP was carried out, then a mixture of steroid and local anesthesia was injected. Visual Analog Scales (VAS) for low back pain and leg pain, and complications were evaluated. *Results:* A total of 271 patients with a minimum 6-month follow-up were enrolled. There were 80 patients who had a history of previous lumbar spinal surgery (F group), and 191 patients without previous lumbar spinal surgery (N group). There were no significant differences in sex and age between the two groups. VAS scores for low back pain (N group/F group) preoperatively, immediately postoperatively, and 1 month, 3 months and 6 months postoperatively, were 51/52 mm, 24/26 mm, 33/34 mm, 30/36 mm, and 30/36 mm, respectively. VAS scores for leg pain were 69/67 mm, 28/27 mm, 39/41 mm, 36/43 mm, and 32/40 mm, respectively. Both VAS scores for low back pain and leg pain were significantly decreased from baseline to final follow-up in both groups (*p* < 0.01). However, VAS scores for leg pain at 3 months and 6 months postoperatively were significantly higher in F group (*p* < 0.05). There were three catheter breakages (2/3 in F group), and one dural tear in F group. *Conclusions:* TSCP significantly reduced both VAS scores for low back and leg pain in patients with and without FBSS. However, co-existence of intractable epidural adhesion might be associated with less improvement in FBSS.

## 1. Introduction

The aging of the population will be a worldwide problem. According to the World Health Organization and the United Nations, when the proportion of persons aged 65 years or older exceeds 7% in a society, it is called an aging society. If it exceeds 14%, it is defined as an aged society; and if it exceeds 21%, it is defined as a super-aged society. Nowadays, the elderly population is about 28.4% in Japan, thus it is categorized as a super-aged society, and is the highest in the world [1]. Because clinicians are required to manage an increasing number of elderly patients, and associations between increasing age, increasing operative duration, length of stay, and revision surgery with adverse events have been reported [2], surgical invasiveness should be reduced to avoid perioperative complications, especially in elderly and immunocompromised patients.

To date, spinal surgeries have been increasing and are associated with the development of various surgical techniques and recent spinal instrumentation. However, one of the problems is residual and/or recurrent symptoms after lumbar spinal surgery. North et al. first termed this condition “failed back surgery syndrome (FBSS)” [3]. Chan et al. reported that the incidence of FBSS was 10% to 40% after lumbar spinal surgery [4]. The first-line treatment for FBSS is nonsurgical treatment [5]. If nonsurgical treatment fails, reoperation would be considered. Ideal surgical treatment is not always applied because of the risks of surgical invasiveness in elderly patients, or sometimes patients may refuse major surgery by themselves. Conventional approaches of lumbar spinal surgeries are all from outside of the spinal canal even in minimally invasive spine surgery. In contrast, intraspinal canal treatment (ISCT) is the direct approach to the spinal canal. Percutaneous epidural neuroplasty (PEN) is a minimally invasive lysis procedure releasing the epidural adhesions and injecting steroids, saline, and analgesics through the epidural access catheter [6]. PEN would be a less invasive approach for FBSS in the elderly or immunocompromised patients.

Here, we named our PEN procedure as trans-sacral canal plasty (TSCP) in the sub-study group of minimally invasive spinal treatment (MIST) society (clinical research group). TSCP would be applied to chronic and residual low back pain and/or leg pain due to various lumbar spinal disorders. The aim of this study was to evaluate the efficacy of TSCP between patients with and without FBSS.

## 2. Materials and Methods

**Figure 1:**
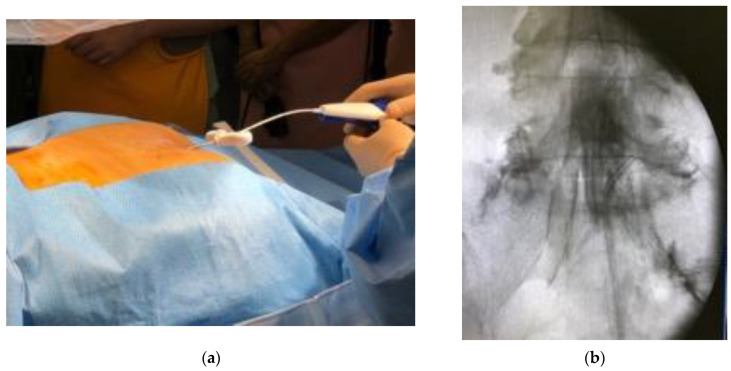
Procedure of trans-sacral canal plasty (TSCP). (**a**) The patient was placed in a prone position, and a local anesthesia was given around the sacral hiatus. The introducer was placed into sacral canal through the sacral hiatus under fluoroscopic guidance. The epidural access catheter was inserted through the introducer after removal of the inner cylinder. (**b**) Contrast dye was injected into epidural space in order to confirm exact epidural placement of the catheter, and to identify any filling defects suggestive of pathological lesion with epidural adhesion. After a catheter was placed at lesion site, an adhesiolysis was carried out by both moving the catheter for mechanical lysis and injecting normal saline for liquid lysis.

**Figure 2:**
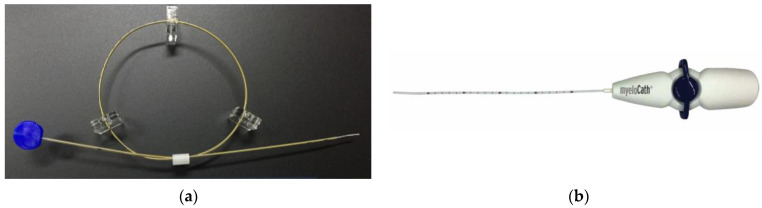
Epidural access catheter. (**a**) Racz catheter (BREVI-XL™, Epimed International Inc., Dallas, TX, USA). (**b**) Epiduroscopy catheter (myeloCath^®^, Biomedica Healthcare Ltd., Tokyo, Japan).

A multicenter retrospective analysis from seven facilities was conducted. TSCP was performed in the patients who suffered from chronic low back pain and leg pain due to lumbar spinal disorders. Contraindications are coagulation abnormality, pregnancy, contrast allergy, infections or tumor in the spinal lesion. The patient was placed in a prone position. Vital signs, including electrocardiogram, blood pressure and pulse oximeter were monitored. After local anesthesia was given around the sacral hiatus, the introducer was placed into sacral canal through the sacral hiatus under fluoroscopic guidance, then the epidural access catheter was inserted through the introducer after the removal of the inner cylinder (Figure 1a). A Racz catheter (BREVI-XL™, Epimed International Inc., Dallas, TX, USA) or epiduroscopy catheter (myeloCath^®^, Biomedica Healthcare Ltd., Tokyo, Japan) was used as an epidural access catheter (Figure 2). Contrast dye was injected into the epidural space in order to confirm the exact epidural placement of the catheter, and to identify any filling defects suggestive of a pathological lesion with epidural adhesion (Figure 1b). After a catheter was placed in the epidural space, an adhesiolysis was carried out by both moving the catheter for mechanical lysis and injecting normal saline for liquid lysis. Then, a mixture of steroid and lidocaine/bupivacaine was injected. Visual Analog Scales (VAS) for low back pain and leg pain were evaluated preoperatively, immediately postoperatively, and 1 month, 3 months and 6 months postoperatively. Perioperative complications were also assessed.

All data were expressed as the mean ± standard deviation. The VAS scores preoperatively, immediately postoperatively, and 1 month, 3 months and 6 months postoperatively were compared between N group and F group using an independent *t*-test. A Chi-square test was also used to compare patients’ demographics and outcomes. A *p*-value < 0.05 was considered statistically significant.

## 3. Results

A total of 271 patients with a minimum 6-month follow-up were enrolled. Diagnosis consisted of lumbar spinal stenosis (240), degenerative spondylolisthesis (27), lumbar disc disease (4), lumbar disc herniation (19), degenerative lumbar scoliosis or kyphosis (13), osteoporotic vertebral fracture (5), iatrogenic (3), and unknown reason (11), and 56 patients were diagnosed with several diseases. There were 191 patients without previous lumbar spinal surgery (N group: male 87/female 107, ave. 70.2 ± 11.9 years old), and 80 patients who had a history of lumbar spinal surgery (F group: male 37/female 43, ave. 72.7 ± 10.2 years old). The majority of the patients were in their 60s to 80s (228/271 = 84.1%). There were no significant differences in sex and age between N group and F group (*p* = 0.789, *p* = 0.103). The Racz catheter was more frequently used in N group (65/191 = 34.0%) than in F group (16/80 = 20.0%) (*p* < 0.05).

**Figure 3:**
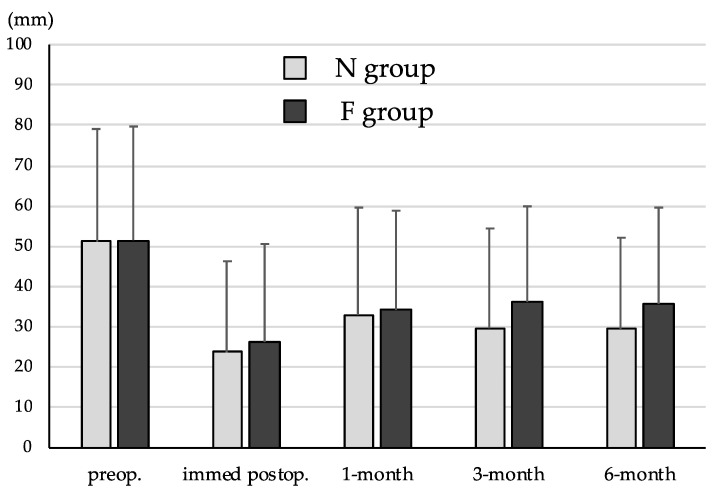
Visual Analog Scales (VAS) scores for low back pain. VAS scores for low back pain were significantly decreased from baseline to final follow-up in both groups (*p* < 0.01). VAS scores for low back pain 3 months and 6 months postoperatively tended to be higher in F group.

**Figure 4:**
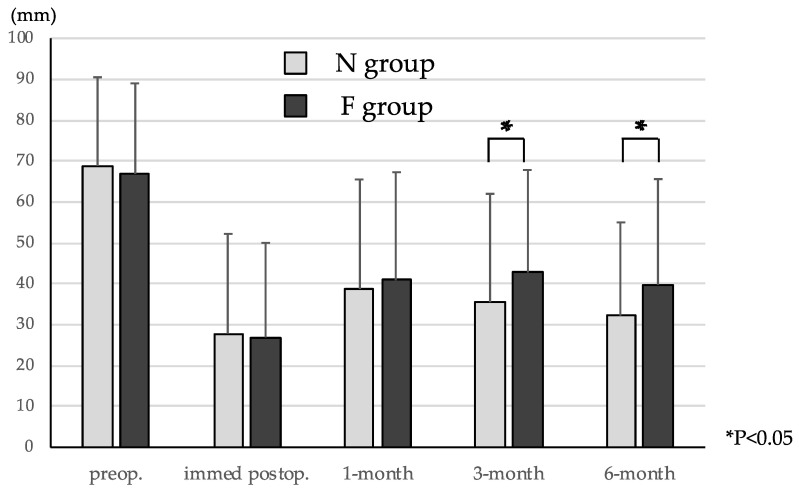
VAS scores for leg pain. VAS scores for leg pain were significantly decreased from baseline to final follow-up in both groups (*p* < 0.01 each). However, VAS scores for leg pain 3 months and 6 months postoperatively were significantly higher in F group (*p* < 0.05 each).

VAS scores for low back pain in N group preoperatively, immediately postoperatively, and 1 month, 3 months and 6 months postoperatively were 51.1 ± 28.0 mm, 23.7 ± 22.6 mm, 33.0 ± 26.6 mm, 29.7 ± 24.7 mm, and 29.5 ± 22.6 mm, and those in F group were 51.5 ± 28.2 mm, 26.3 ± 24.3 mm, 34.2 ± 24.6 mm, 36.2 ± 23.7 mm, and 35.7 ± 23.9 mm, respectively (Figure 3). VAS scores for leg pain in N group preoperatively, immediately postoperatively, and 1 month, 3 months and 6 months postoperatively were 68.8 ± 21.7 mm, 27.8 ± 24.4 mm, 38.7 ± 26.8 mm, 35.5 ± 26.5 mm, and 32.2 ± 22.8 mm, and those in F group were 66.9 ± 22.1 mm, 26.9 ± 23.1 mm, 40.9 ± 26.4 mm, 42.8 ± 25.0 mm, and 39.8 ± 25.8 mm, respectively (Figure 4). Both VAS scores for low back pain and leg pain were significantly decreased from baseline to final follow-up in both groups (*p* < 0.01 each). However, VAS scores for leg pain 3 months and 6 months postoperatively were significantly higher in F group (*p* < 0.05 each). Although VAS scores for low back pain 3 months and 6 months postoperatively tended to be higher in F group, they did not reach a significance. A more than 50% reduction of the VAS score for low back pain 3 months postoperatively was obtained in 71/191 patients (37.2%) in N group, and in 25/80 patients (31.3%) in F group. A more than 50% reduction of the VAS score for leg pain 3 months postoperatively was obtained in 89/191 patients (46.6%) in N group, and in 26/80 patients (32.5%) in F group. Although there was no significant difference between the two groups in low back pain, leg pain significantly remained in F group (*p* < 0.05).

Overall, definitive surgery was performed in 69/271 patients (32.1%) until final follow-up due to residual low back/leg pain after TSCP, or neurological deterioration. Definitive surgery was performed more frequently in N group (69/191 = 36.1%) than in F group (18/80 = 22.5%) (*p* < 0.05). Decompression surgery was performed more frequently in N group (48/191 = 25.1%) than in F group (7/80 = 8.8%) (*p* < 0.01). Fusion surgery was performed equally in N group (21/191 = 11.0%) and in F group (11/80 = 13.8%).

There were 3 catheter breakages, and 2/3 cases were observed in F group, and one dural tear with transient neurological deficit occurred in F group. No major complications were observed during the follow-up period.

## 4. Discussion

Spinal surgeries have been increasing and are associated with the development of various surgical techniques and spinal instrumentation. However, one of the problems is residual or recurrent low back pain and/or leg pain after lumbar spinal surgery, which has been recognized as FBSS [3]. Recurrent lumbar disc herniation, restenosis, postoperative instability, adjacent segment disease, or instrumentation failure are representative causes as postoperative factors. The first-line treatment for FBSS without neurological deficit or severe instability is nonsurgical treatment including medication, nerve block, bracing, cognitive behavioral therapy, rehabilitation, and so on. Nowadays, spinal cord stimulation is applied to FBSS for neuromodulation [7]. When nonsurgical treatment fails, repetitive surgical treatments would be considered. If recurrent lumbar herniation occurs, herniotomy or instrumented fusion would be performed. If the patients develop global malalignment after adult deformity surgery, major revision surgery would be performed for definitive surgical treatment [8]. However, multiple spinal surgeries are sometimes inadvisable in the elderly or immunocompromised patients. Ideal surgical treatment is not always applied because of the risks of surgery, or sometimes patients may refuse invasive surgical treatments by themselves.

ISCT is the direct approach to the spinal canal, and PEN is a minimally invasive lysis procedure for chronic low back pain and/or leg pain due to lumbar spinal disorders [6]. The myeloscope and epidural approach has been developed since the 1930s. Burman first used arthroscopic equipment for the assessment of spinal pathology in a cadaveric study [9]. Pool developed a rigid endoscope for first clinical use and assessed normal and pathological conditions in 400 cases [10]. Blomberg found the dorsomedian connective tissue connecting dura and ligamentum flavum [11]. Shimoji et al. used a flexible and steerable epiduroscope and identified the affected nerve root and reproduced the pain by touching [12]. The myeloscope had mostly been used as a diagnostic tool until the development of magnetic resonance imaging. Recently, Racz developed a lysis technique of epidural adhesions for radicular and/or low back pain [13]. Saberski also reported an epiduroscopy technique with lysis procedure for residual pain after an epidural block [14]. Several randomized control studies of PEN were conducted and showed a significant effect, especially in pain control [15,16]. PEN had better pain relief compared to epidural injections due to the lysis procedure that improved the spread of medications [16]. Previous studies have reported the efficacy of PEN for FBSS patients. Avellanal et al. reported on 24 patients, and they mentioned that 54% of patients obtained >50% pain reduction [17] Ceylan et al. also reported on 82 patients with FBSS, and they mentioned that VAS scores and the oswestry disability index significantly reduced after epiduroscopy, and epiduroscopy was more effective in FBSS patients without stabilization (without spinal instrumentation) than FBSS patients with stabilization (with spinal instrumentation). They also found that epiduroscopy was useful for accurate diagnosis [18].

**Figure 5:**
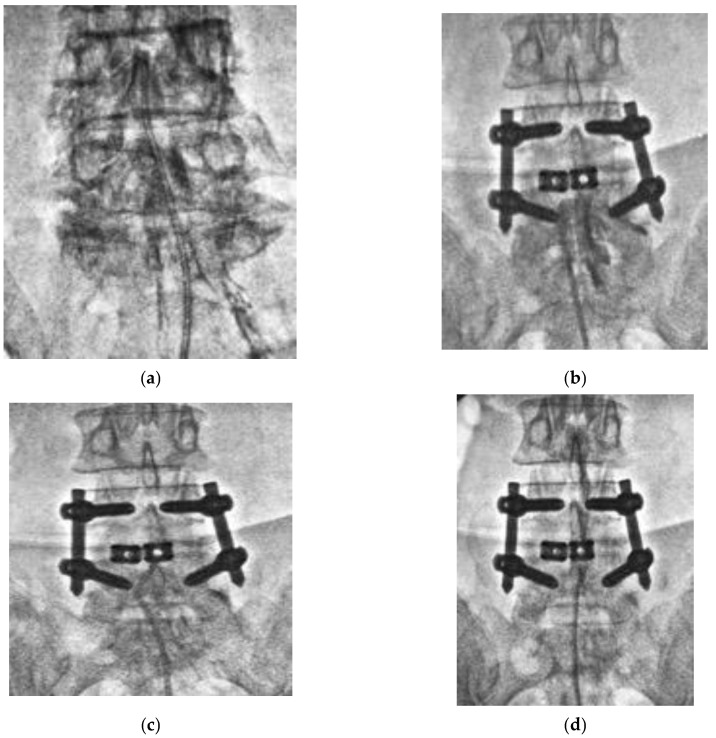
Epidurogram during TSCP procedure. (**a**) Epidurogram of a patient without any history of lumbar spinal surgery. The catheter was inserted into the epidural space with a mild to moderate resistance, and a spread of contrast dye seemed to be diffuse. (**b**) Epidurogram of a patient with a history of previous lumbar spinal surgery. The catheter was stuck, and contrast dye did not spread at the site of adhesion. (**c**) Breakage of tip of the catheter occurred due to severe adhesion in the epidural scape. (**d**) The distribution of contrast dye seemed to be poor.

To date, there has been no comparative study investigating the efficacy of PEN between patients with and without FBSS. We named our lysis procedure TSCP and conducted a comparative study investigating the efficacy of TSCP between patients with and without FBSS in a multicenter analysis. Our results showed that TSCP significantly reduced low back pain and leg pain both in patients with and without FBSS. In this study, the majority of the patients were in their 60s to 80s. Before we could perform the TSCP procedure, we had to consider definitive surgery, even for the elderly patients, in case nonsurgical treatment failed. However, some of the elderly patients could not undergo general anesthesia due to their medical conditions. TSCP would be a less invasive approach for the elderly or immunocompromised patients under local anesthesia. However, leg pain significantly remained in FBSS patients 3 months and 6 months after TSCP. There might be a severe epidural adhesion in FBSS patients due to previous surgeries. The catheter was stuck, and contrast dye did not spread effectively at the site of adhesion (Figure 5b), and breakage of the tip of the catheter could occur (Figure 5c). Even when the contrast dye went into the cranial side, the distribution of dye seemed to be poor (Figure 5d); thus, the analgesic effect might be less in FBSS patients compared to patients without any history of lumbar spinal surgery (Figure 5a). There were three catheter breakages, and two of those cases were observed in FBSS, and one dural tear with transient neurological deficit occurred in FBSS patients. These complications might be related to severe adhesion in FBSS. Fortunately, there was no permanent neurological deficit or major complication perioperatively in this study. Marchesini et al. [19] reported that the complication rate of PEN was 8% in their review of 244 patients, and they found that more than half of complications were dural tears, and 93% of dural tears were observed in FBSS patients. Non-persistent post-procedural low back and/or leg discomfort/pain, transient neurological symptoms related to the increase in intracranial pressure, infection, post-procedural visual impairment with retinal hemorrhages, intravascular injection, encephalopathy resulting in rhabdomyolysis due to a dural tear, and neurogenic bladder and seizures were also reported as other perioperative complications. Avellanal et al. had proposed a “60 limit rule” for the prevention of an increase in intracranial pressure; procedures should not last more than 60 min, not exceed 60 mL of injection, and not exceed 60 mmHg of epidural pressure [20].

Our study showed that definitive surgery was performed in 32.1% due to residual pain after TSCP, and definitive surgery and decompression were more frequently performed in N group. We suggested that patients with FBSS might be prone to refusing reoperation even if they have residual leg pain. Although TSCP significantly reduced low back pain and leg pain in both patients with and without FBSS, the TSCP procedure is still under development, and it is needed to achieve better clinical outcomes by improving the lysis procedure and developing equipment which can be available in the epidural space in the future.

## 5. Conclusions

TSCP was considered a minimally invasive and safe procedure, and it significantly reduced both VAS scores for low back and leg pain in patients with and without FBSS. It can be a treatment option for elderly and immunocompromised patients, for whom multiple surgeries are inadvisable. However, the co-existence of intractable epidural adhesion might be associated with less improvement in leg pain for patients with FBSS. Dural tear and catheter breakage should be avoided in FBSS.

## Data Availability

Not applicable.
